# Revaluation of the tongue function of the modified hypoglossal-to-facial transfer: a comparative cohort study

**DOI:** 10.3389/fneur.2026.1738616

**Published:** 2026-09-21

**Authors:** Ying Zhang, Zhongding Zhang, Yinda Tang, Baimiao Wang, Xiaomin Cai, Yuhan Li, Xianda Wu, Luonan Ni, Gongqiang Li, Yunling Jiang, Hua Zhao, Lifeng Wang, Jin Zhu, Shiting Li

**Affiliations:** 1 Department of Neurosurgery, Xinhua Hospital Affiliated to Shanghai Jiao Tong University School of Medicine, Shanghai, China; 2 Department of Pathology, Xinhua Hospital Affiliated to Shanghai Jiao Tong University School of Medicine, Shanghai, China; 3 Neurological Disease Prevention and Treatment Center, Yanjin County People's Hospital, Yanjin, Yunnan Province, China

**Keywords:** descendens hypoglossi, facial palsy, functional neurosurgery, hypoglossal-facial nerve anastomosis, tongue function

## Abstract

**Background and objectives:**

Traditional end-to-end hypoglossal-to-facial anastomosis (HFA) was a common surgical procedure for uncured severe facial palsy, however, was related to tongue dysfunction. We developed a modified hypoglossal-to-facial anastomosis (MHFA) procedure with the additional process of the anastomosis of the transected hypoglossal nerve and the ramus descendens hypoglossi, aiming to salvage the tongue function.

**Methods:**

We retrospectively collected 45 cases of MHFA in our center from January 2020 to December 2022. A comparative analysis was conducted from MHFA and 27 cases of HFA in the early stage at our center. The tongue disability index was used to evaluate the tongue function during the follow-up.

**Results:**

Baseline characteristics were comparable between HFA (*n* = 27) and MHFA (*n* = 45) groups (all *p* > 0.05). MHFA demonstrated longer operative time (198.27 vs. 157.78 min, *p* < 0.001). Up to the last follow-up, MHFA showed superior outcomes: smaller tongue deviation angle (13.38° ± 9.56° vs. 23.86° ± 10.52°, mean difference 10.47°[95%CI 5.65–15.29], *p* < 0.001), lower incidence of severe deviation (>30°: 8.9% vs. 37.0%, *p* = 0.003), and reduced tongue-related complications (24.4% vs. 66.7%, *p* < 0.001). Specifically, MHFA had significantly fewer swallowing (11.1% vs. 37.0%, *p* = 0.009) and articulation defects (13.3% vs. 44.4%, *p* = 0.003). TDI scores were markedly lower in MHFA (1.63 ± 0.53 vs. 2.23 ± 0.50, mean difference 0.60 [0.34–0.85], *p* < 0.001), particularly in swallowing, articulation, and psychological subdomains (all *p* < 0.05). Strong correlation existed between tongue deviation and TDI scores (*r* = 0.890, *p* < 0.001). Facial nerve recovery showed no intergroup difference (HB score improvement: 2.40 ± 0.69 vs. 2.33 ± 0.73, *p* = 0.699).

**Conclusion:**

MHFA appears promising for reducing tongue morbidity, but its clinically relevant advantage over HFA requires prospective validation using standardized outcomes.

## 1 Introduction

Facial paralysis significantly impacts patients’ quality of life and physical-mental health, with severe cases of House-Brackmann grades V-VI often requiring surgical intervention (1–3). Surgical approaches vary depending on etiology, disease course, severity, and patient preferences. Facial nerve substitution grafting has become a prevalent option, particularly suitable for patients with proximal (central) facial nerve damage while maintaining intact distal nerve segments and functional neuromuscular junctions in facial muscles (4). Common donor nerves include the hypoglossal, accessory, and masseteric nerves, with Hypoglossal-Facial Anastomosis (HFA) being the most widely used technique (5–7). The conventional HFA procedure involves complete transection of the distal hypoglossal nerve followed by end-to-end anastomosis with the extracranial main trunk of the facial nerve. This approach offers distinct advantages: (1) ample nerve fiber supply from the hypoglossal trunk; (2) comparable diameter between donor and recipient nerves; (3) anatomical proximity facilitating surgical access (4) demonstrated efficacy in facial function recovery (8–10). However, complete hypoglossal nerve transection inevitably causes ipsilateral tongue dysfunction, potentially leading to tongue hemi-atrophy and significant impairment tongue function including mastication, swallowing, and speech (11). This inherent limitation has driven ongoing research efforts to refine traditional HFA techniques, aiming to preserve tongue function while maintaining optimal facial reanimation outcomes—a crucial focus in contemporary facial nerve rehabilitation studies.

To address the limitation of traditional HFA, various modified techniques have been proposed, including nerve grafting, hemihypoglossal nerve transfer (end-to-side anastomosis), and split anastomosis (5, 12–14). However, these approaches face challenges such as insufficient donor nerve fibers and suboptimal facial function recovery. As early as 1979, Conley and Baker proposed direct anastomosis between the ansa cervicalis and facial nerve, but subsequent reports demonstrated that postoperative recovery only reached a maximum HB grade IV, likely due to inadequate fiber quantity in the ansa cervicalis (15, 16). Recent studies have proposed selecting candidates for facial-descendens hypoglossi anastomosis based on the diameter of the descendens hypoglossi nerve, however, the functional outcomes still fall short of those achieved with conventional hypoglossal-facial anastomosis (17). Building on previous findings, our team has developed a modified hypoglossal-to-facial anastomosis (MHFA) procedure, combined hypoglossal-facial and cervical descendens hypoglossi-hypoglossal anastomoses, which combined conventional HFA with anastomosis of the descendens hypoglossi (superior root of the ansa cervicalis derived from cervical nerves) to the distal hypoglossal trunk (18). Our preliminary prospective small-sample study has demonstrated that MHFA maintains the proven efficacy of hypoglossal-facial anastomosis while effectively preserving tongue function through descendens hypoglossi supplementation (19). The present study aimed to evaluate tongue-related and facial outcomes after MHFA in a larger, nonoverlapping retrospective cohort and to determine whether the findings of our previous study remained consistent over longer follow-up.

## 2 Method

### 2.1 Patients

We retrospectively analyzed the clinical data patients with severe peripheral facial palsy who underwent nerve substitution surgery between January 2020 and October 2023. A total of 104 patients were retrieved from the hospital information system. To prevent bias introduced by repeated analyses, 20 cases that had been included in our previous prospective study during the time period were excluded (19). Besides, two patients were excluded for incomplete clinical records, and 4 patients were excluded for loss of follow-up. Finally, 27 cases of traditional hypoglossal-to-facial anastomosis (HFA) and 45 cases of modified hypoglossal-to-facial anastomosis (MHFA) were enrolled in the present study. All procedures are performed by the same surgical team.

All patients who underwent surgery had House-Brackmann (HB) grades of V or VI. Preoperative CT and MRI were performed for each patient to exclude intracranial lesions (new-found or recurrent tumors, infarction, hemorrhage, etc.). We began to shift to perform MHFA in 2020 gradually. The cases of traditional HFA mainly include patients during/before surgical procedure shift, and patients whose descendens hypoglossi were not visible or its length did not allow for the anastomosis with the transected hypoglossal nerve. The follow-up period ranged from 12 to 42 months, and the mean follow-up period was 24.75 months. The written informed consent was obtained from each patient before surgery. The study was approved by the ethics committee of our hospital.

### 2.2 Surgical procedure

MHFA surgery starts by making a 6–8 cm curved incision behind the ear, from the mastoid tip to about 1.5 cm below the angle of the mandible. Then the tissue and muscle layers are carefully dissected. The extracranial segment of the facial nerve is exposed at the stylomastoid foramen, the hypoglossal nerve is revealed deep to the posterior belly of digastric muscle, and retrograde dissection is conducted along the hypoglossal nerve trunk to locate and isolate the descendens hypoglossi extending to its distal end for later use (Figure 1A). Subsequently, the previously exposed hypoglossal nerve, descendens hypoglossi, and main trunk of the extracranial part of the facial nerve are transected (Figure 1B). The distal end of the hypoglossal nerve is anastomosed to the proximal end of the descendens hypoglossi (Figure 1C), while the proximal end of the hypoglossal nerve is anastomosed to the distal end of the facial nerve (Figure 1D). Nerve sheaths (GI 5 × 30, TianXinFu Medical Appliance Co., Ltd.) are wrapped around the anastomosis site for protection, and it is secured with human fibrin glue. Layers of muscle and skin are then sutured step by step, concluding the surgery.

**Figure 1:**
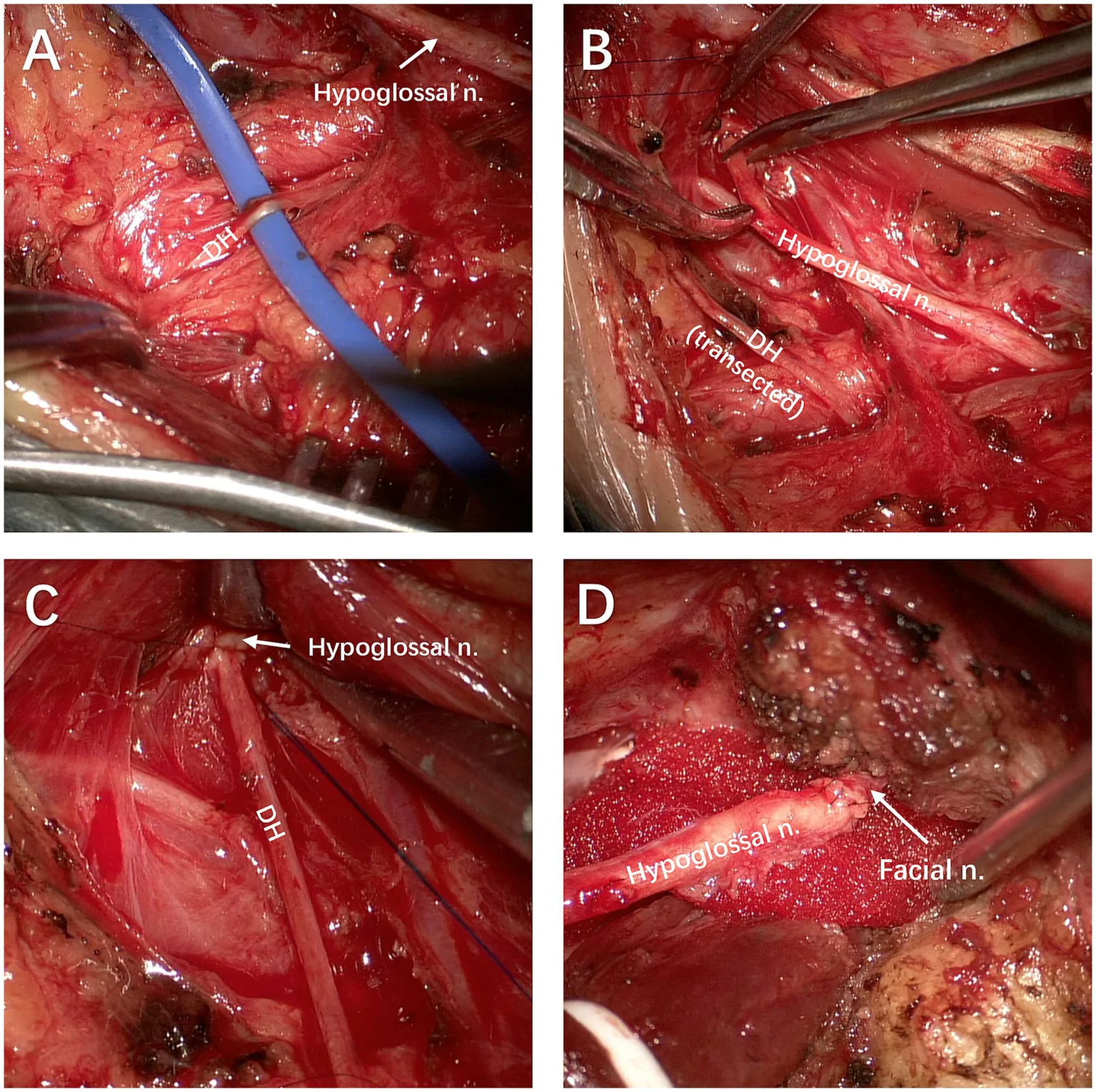
Intraoperative procedure of modified hypoglossal-to-facial transfer. **(A)** Retrograde dissection is conducted along the hypoglossal nerve trunk to locate and isolate the Descendens hypoglossi. **(B)** Descendens hypoglossi and hypoglossal nerve were transected sequentially. **(C)** The proximal end of Descendens hypoglossi is anastomosed to the distal end of the hypoglossal nerve. **(D)** The proximal end of the hypoglossal nerve is anastomosed to the distal end of the facial nerve. DH, Descendens hypoglossi; n., nerve.

Traditional HFA does not contain the steps of locating, transecting, and anastomosing the descendens hypoglossi with the hypoglossal nerve, while the other steps are the same as the RHRT. Patients in both groups received neurotrophic drug therapy and facial rehabilitation training after surgery.

After the operation, both groups of patients received the same standard postoperative care. Both groups of patients were prescribed mecobalamin (0.5 mg, tid) and vitamin B1 (20mg, tid) to nourish the nerves. The medication duration for both groups was 12 months. And once the patient’s surgical incision has healed well, the tongue muscle function rehabilitation training can be initiated. The training content is as follows: Ask the patient to extend the tongue as far forward, to the left, and to the right as possible. Each movement should be maintained for about 5 s before returning to the natural position. Each set of these movements should be repeated 20 times, and the training should be conducted 3 times a day. Before the patient is discharged, they should complete the relevant rehabilitation exercises in a standardized manner under the guidance of the doctor, and it is recommended that they continue to perform tongue muscle function exercises after discharge for a period of at least 12 months.

### 2.3 Outcomes evaluation

Patients were asked to return to the hospital for follow-up at 3, 6, 9, and 12 months after surgery. Due to the absence of follow-up in some patients, all patients were investigated again by telephone and Internet between September 2023 and March 2024. Videos of facial and tongue movements were obtained to assess the degree of recovery from facial paralysis and tongue dysfunction. Patients were instructed to protrude their tongue as far as possible and in the midline direction, with maximal effort, to standardize the assessment. Each patient was asked to perform the tongue protrusion three times, and the best attempt (i.e., the one with the most forward extension and minimal lateral deviation) was photographed. The same standardized verbal instructions were given by the same examiner for all patients to ensure consistency. Photographs of the extended tongue were obtained to measure the tongue deviation angle (Figure 2). Tongue disability index (TDI), a self-administered questionnaire, was used to evaluate tongue function including subjective feelings of chewing difficulty, swallowing defect, articulation defect, and their impact on psychology (Table 1) (19). The questionnaire was acquired by online questionnaire applet or by telephone interview with patients’ oral description. Facial nerve function was assessed using both the House-Brackmann (HB) grading system and the Sunnybrook Facial Grading System. The Sunnybrook composite score was calculated as the voluntary movement score minus the resting symmetry penalty and synkinesis score (range, 0–100; higher scores indicate better facial function).

**Figure 2:**
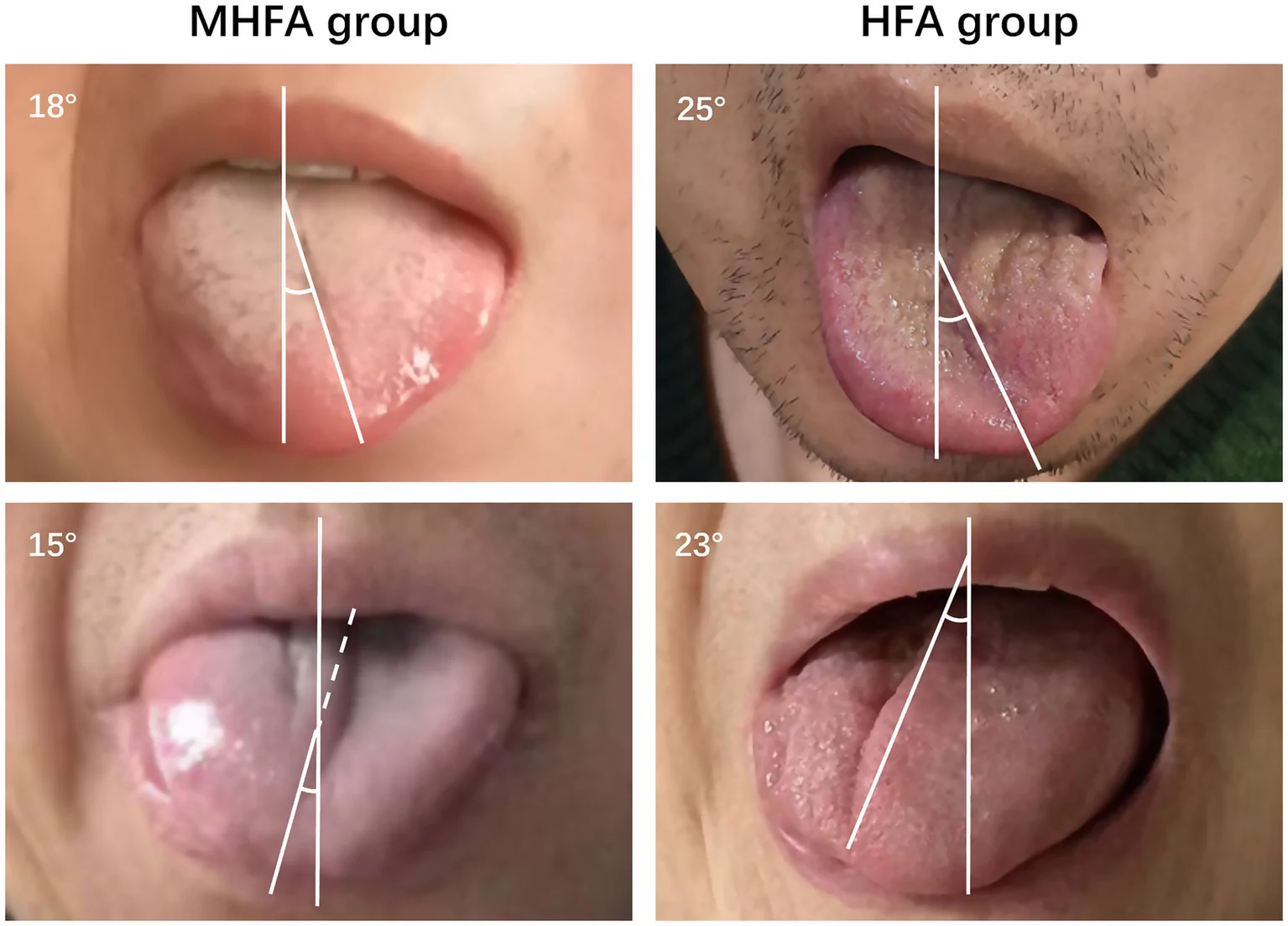
Measurement of the tongue deviation angle. The evaluator determines the tongue deviation angle by measuring the angle between the midline of the tongue and the facial midline in photographs or video screenshots captured when the patient’s tongue is at its maximum extended position.

**Table 1:** Tongue disability index (TDI).

Score	Four domains			
	Chewing difficulty	Swallowing defect	Articulation defect	Psychological impact
1	Completely normal function			No impact
2	Relatively intact function but uneasy feeling			Self-consciousness but no impact on daily social activity
3	Substantially compromised function			Gross impact on daily social activity
4	Complete loss of the function			Complete avoidance of social activity

TDI score is the average of the 4 sub-scores.

The primary outcome was the tongue deviation angle at the last follow-up. The TDI score was considered a key secondary outcome. Other secondary outcomes included tongue-related symptoms, and facial nerve recovery assessed using the HB and Sunnybrook grading systems.

### 2.4 Data analysis

Data analyses were performed using SPSS 25.0 (IBM Corp., Armonk NY, USA) and python 3.40. The Kolmogorov–Smirnov test was conducted to examine the normality of variables. For continuous numerical variables, Students’ t test or Mann–Whitney U test was used to compared the differences between groups, as appropriate. For categorical variables, chi-square test, continuous correction chi-square test, or Fisher exact tests were used for analysis, as appropriate. For ordinal variables, Mann–Whitney U test was used for comparison. Pearson linear correlation was employed in analysis of the relation between the TDI score and the tongue deviation angle. Multivariable linear regression with HC3 robust standard errors was used for continuous outcomes, adjusting for age, sex, log2-transformed symptom duration, and follow-up duration. The postoperative Sunnybrook model additionally adjusted for baseline score. Binary outcomes were analyzed using Firth logistic regression because of limited event counts. Adjusted mean differences or odds ratios are reported with 95% confidence intervals. All tests were two-sided, and *p* < 0.05 was considered statistically significant.

## 3 Result

The baseline characteristics of patients are presented in Table 2. The mean age was 47.44 years in the HFA group and 49.69 in the MHFA group (*p* = 0.502). The sex (*p* = 0.715), side (*p* = 0.670), causes (*p* = 0.0.562), symptoms duration (*p* = 0.836), preoperative HB score (*p* = 0.508), Sunnybrook score (*p* = 0.893) and follow-up times (*p* = 0.079) were comparable between two groups. The mean operating time was 157.78 min in the HFA group versus 198.27 min in the MHFA group (*p* < 0.001). No intraoperative injury or postoperative incision-related complications were encountered during surgery in either group.

**Table 2:** Baseline characteristics of HFA group and MHFA group.

Characteristic	HFA (*n* = 27)	MHFA (*n* = 45)	*p* value
Age (years)	47.44 ± 14.73	49.69 ± 11.60	0.502
Sex			0.715
Male	12	22	
Female	15	23	
Site			0.670
Left	13	24	
Right	14	21	
Causes^a^			0.562
CPA Tumor resection	22	39	
Trauma	4	6	
Viral infection	1	0	
Time from onset to surgery (months)	21.81 ± 27.46	19.94 ± 41.78	0.836
Preop HB score			0.508
V	23	37	
VI	4	8	
Preop Sunnybrook score	9.07 ± 5.70	9.27 ± 6.14	0.893
Operating time (minutes)	157.78 ± 31.48	198.27 ± 39.37	<0.001
Follow-up times (months)	27.19 ± 9.49	23.29 ± 8.67	0.079

HFA, Hypoglossal-to-facial anastomosis; MHFA, Modified hypoglossal-to-facial anastomosis; CPA, cerebellopontine angle; HB, House-Brackmann.

a Fisher–Freeman–Halton test was used for comparison.

All patients were followed for more than 1 year. At the last follow-up, the mean tongue deviation angle was 23.86° ± 10.52° in the HFA group versus 13.38° ± 9.56° in the MHFA group (mean difference 10.47°, 95% CI 5.65 to 15.29; *p* < 0.001). The tongue deviation angle was > 30° in 10 cases (37.0%) of patients in the HFA group and 4 cases (8.9%) of the patients in the MHFA group (*p* = 0.003). The TDI score in MHFA group was significantly lower (2.23 ± 0.50 in HFA group vs. 1.63 ± 0.53 in MHFA group, mean difference 0.60, 95% CI 0.34 to 0.85; *p* < 0.001). Besides, the sub-score of TDI including Swallowing (*p* < 0.001), Articulation (*p* < 0.001) and Psychological Impact (*p* = 0.035) were significantly lower in the MHFA group. After adjustment for age, sex, symptom duration, and follow-up duration, MHFA remained associated with a smaller tongue deviation angle (adjusted mean difference, −11.03°; 95% CI, −16.10 to −5.97; *p* < 0.001) and a lower TDI score (adjusted mean difference, −0.64; 95% CI, −0.90 to −0.38; *p* < 0.001). MHFA was also associated with lower odds of severe tongue deviation (adjusted OR, 0.13; 95% CI, 0.03–0.56; *p* = 0.006) and any tongue-related symptom (adjusted OR, 0.12; 95% CI, 0.03–0.41; *p* < 0.001; Table 3).

**Table 3:** Unadjusted and multivariable-adjusted surgical outcomes.

Outcomes	HFA (*n* = 27)^a^	MHFA (*n* = 45)^a^	Unadjusted effect (95% CI)	*p* value	Adjusted effect^b^ (95% CI)	*p* value
Tongue deviation angle, degrees	23.85 ± 10.52	13.38 ± 9.56	MD 10.47 (5.65 to 15.29)	<0.001	aMD 11.03 (5.97 to 16.10)	<0.001
Severe tongue deviation (>30°), n (%)	10 (37.0%)	4 (8.9%)	OR 6.03 (1.66 to 21.90)	0.003	aOR 7.53 (1.77 to 32.00)	0.006
Any tongue-related symptom, n (%)	18 (66.7%)	11 (24.4%)	OR 6.18 (2.16 to 17.67)	<0.001	aOR 8.57 (2.42 to 30.35)	<0.001
Chewing difficulty	7 (25.9%)	6 (13.3%)	OR 2.27 (0.67 to 7.68)	0.304	—	—
Swallowing defect	10 (37.0%)	5 (11.1%)	OR 4.71 (1.40 to 15.85)	0.009	—	—
Articulation defect	12 (44.4%)	6 (13.3%)	OR 5.20 (1.65 to 16.37)	0.003	—	—
Tongue disability index (TDI) score						
Chewing	2.00 ± 0.73	1.69 ± 0.70	MD 0.31 (−0.04 to 0.66)	0.078	—	—
Swallowing	2.33 ± 0.55	1.51 ± 0.69	MD 0.82 (0.51 to 1.14)	<0.001	—	—
Articulation	2.44 ± 0.64	1.71 ± 0.69	MD 0.73 (0.41 to 1.06)	<0.001	—	—
Psychological impact	2.07 ± 0.87	1.62 ± 0.86	MD 0.45 (0.03 to 0.87)	0.035	—	—
TDI mean score^c^	2.23 ± 0.50	1.63 ± 0.53	MD 0.60 (0.34 to 0.85)	<0.001	aMD 0.64 (0.38 to 0.90)	<0.001
Postoperative HB grade, n (%)			—	0.799	—	—
II	10 (37.0%)	18 (40.0%)	—	—	—	—
III	13 (48.1%)	21 (46.7%)	—	—	—	—
IV	3 (11.1%)	4 (8.9%)	—	—	—	—
V	1 (3.7%)	2 (4.4%)	—	—	—	—
Poor facial recovery (HB IV–V), n (%)	4 (14.8%)	6 (13.3%)	OR 1.13 (0.29 to 4.43)	1.000	aOR 1.15 (0.30 to 4.44)	0.840
Mean postoperative HB grade	2.81 ± 0.79	2.78 ± 0.79	MD 0.04 (−0.35 to 0.42)	0.848	—	—
Postoperative Sunnybrook score	66.04 ± 15.78	62.89 ± 14.29	MD 3.15 (−4.33 to 10.63)	0.401	aMD 3.38 (−4.96 to 11.72)	0.420
Decrease in HB grade from baseline	2.33 ± 0.73	2.40 ± 0.69	MD −0.07 (−0.41 to 0.28)	0.699	—	—
Change in Sunnybrook score from baseline	56.96 ± 15.58	53.62 ± 13.69	MD 3.34 (−3.98 to 10.66)	0.363	aMD 3.38 (−4.96 to 11.72)	0.420

a Data are mean ± standard deviation or n (%). Effects compare HFA with MHFA and are reported as mean differences (MDs) for continuous outcomes and odds ratios (ORs) for binary outcomes.

b Adjusted mean differences (aMDs) used HC3-robust linear regression and adjusted odds ratios (aORs) used Firth logistic regression. Models adjusted for age, sex, log2(symptom duration + 1), and follow-up duration; Sunnybrook models additionally adjusted for baseline score.

c TDI score is the average of the 4 domains including chewing, swallowing, articulation, and psychological impact.

There were more tongue related complications happened in HFA group at the last follow-up (66.7% in HFA group vs. 24.4% in MHFA group, *p* < 0.001). The number of cases of chewing difficulty, swallowing defect and articulation defect in HFA group was 7 (25.9%), 10 (37.0%) and 12 (44.4%), respectively. The counterpart in MHFA group was 6 (13.3%), 5 (11.1%) and 6 (13.3%) respectively. The incidence of postoperative swallowing defect (*p* = 0.009) and articulation defect (*p* = 0.003) in HFA group were significantly higher than MHFA group. The Pearson’s correlation analysis revealed significant positive correlation between the TDI score and the tongue deviation angle (correlation coefficient = 0.890, *p* < 0.001; Figure 3).

**Figure 3:**
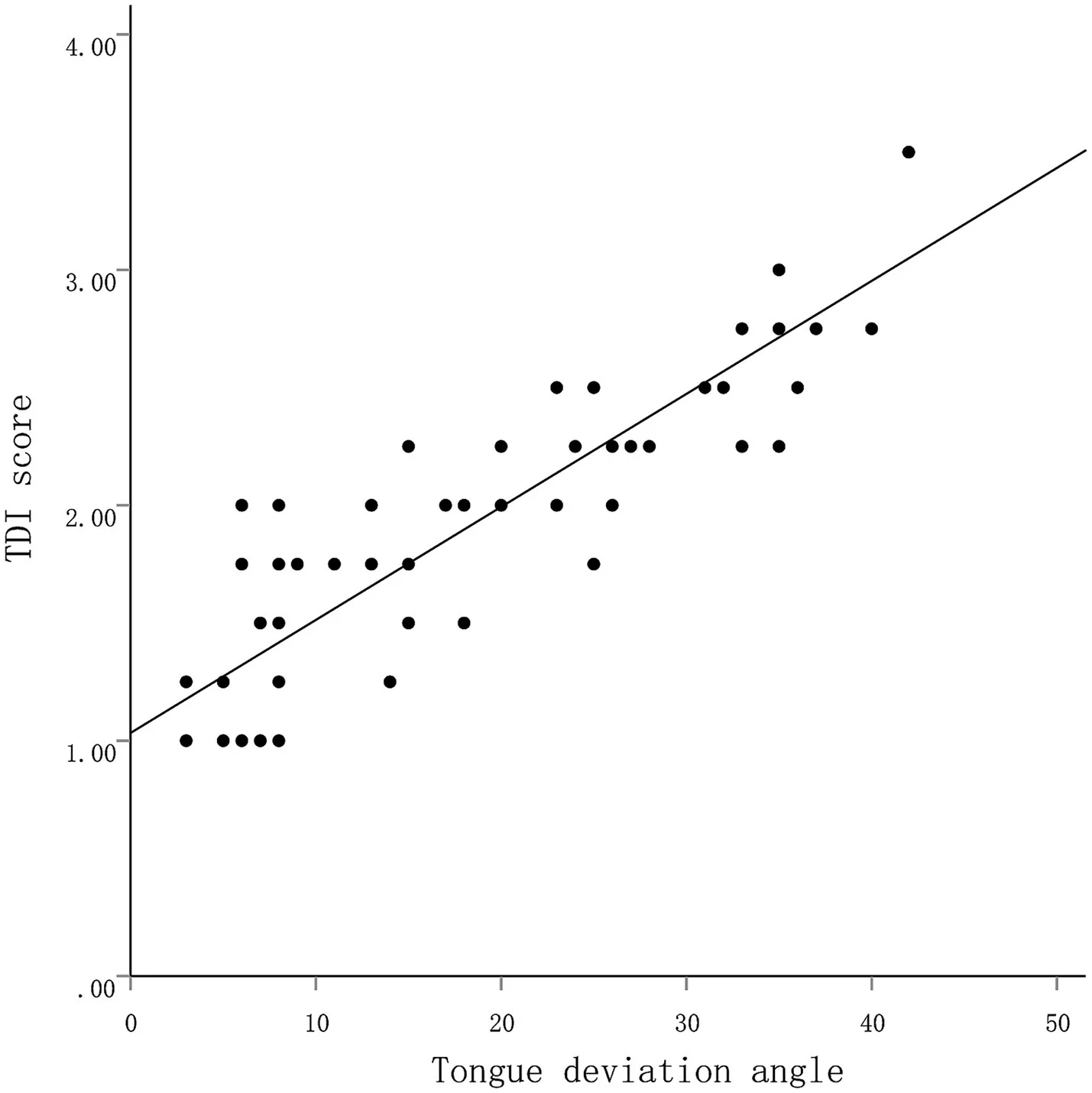
Scatter diagram of TDI score and tongue deviation angle. TDI, tongue disability index.

The facial nerve recovery was comparable between two groups. Twenty-three cases (85.2%) achieving HB grade III or II in the HFA group while 39 cases (86.7%) of counterpart in the MHFA group. The average decrease in HB score from baseline was 2.33 ± 0.73 in HFA group and 2.40 ± 0.69 in MHFA group (mean difference −0.07, 95% CI −0.41 to 0.28, *p* = 0.699). Postoperative Sunnybrook scores were 66.04 ± 15.78 in the HFA group and 62.89 ± 14.29 in the MHFA group, (mean difference, HFA minus MHFA, 3.15; 95% CI, −4.33 to 10.63; *p* = 0.401), and the corresponding changes from baseline were 56.96 ± 15.58 and 53.62 ± 13.69 (mean difference 3.34; 95% CI, −3.98 to 10.66; *p* = 0.363). After adjustment for baseline Sunnybrook score, age, sex, symptom duration, and follow-up duration, the adjusted postoperative difference was 3.38 points (95% CI, −4.96 to 11.72; *p* = 0.420).

## 4 Discussion

Impairment of tongue function is a common complication following HFA. Tongue atrophy has been reported in more than 70% of patients with traditional end-to-end HFA, with the high occurrence rates of dysfunction of speech and swallow (20, 21). Some surgeons have attempted end-to-side anastomosis and split anastomosis techniques to reduce muscle atrophy and preserve tongue function in HFA, but at the expense of axon numbers (22–24). Our comparative analysis demonstrates that our MHFA technique, incorporating descendens hypoglossi anastomosis, significantly mitigated tongue dysfunction compared to classic HFA while maintaining comparable facial nerve recovery. The MHFA group had substantial reduction in tongue deviation angle (13.38° ± 9.56° vs. 23.86° ± 10.52°, *p* < 0.001), lower rates of severe deviation (>30°: 8.9% vs. 37.0%, *p* = 0.003), lower incidence of tongue-related complications (24.4% vs. 66.7%, *p* < 0.001), and lower TDI mean score (1.63 ± 0.53 vs. 2.23 ± 0.50, *p* < 0.001). These findings should be interpreted in the context of other hypoglossal-sparing modifications. Samii et al. (5) reported that direct side-to-end HFA reduced tongue hemiatrophy (5.8% vs. 100%), swallowing difficulty (11.7% vs. 55%), and speech disorder (0% vs. 33%) compared with classic end-to-end HFA, without a significant difference in facial recovery. Martins et al. (25) likewise found lower tongue morbidity after hemihypoglossal-facial neurorrhaphy than after the classic technique, with comparable facial reanimation. Our findings are directionally consistent with these reports, but MHFA differs conceptually because it retains a full end-to-end hypoglossal–facial coaptation while attempting to reinnervate the distal hypoglossal stump through the descendens hypoglossi. The present results also broadly reproduce our previous randomized trial, although the mean tongue deviation angle in the MHFA group was greater with longer follow-up (13.38° vs. 7.8° at 12 months) (19). This difference may reflect time-dependent tongue muscle changes, differences in cohort composition, or measurement variability. Given the heterogeneity in study design, outcome definitions, and follow-up duration, these cross-study comparisons should be regarded as descriptive and do not establish superiority over other modified HFA techniques.

The MHFA group’s superior outcomes in tongue atrophy and swallowing or articulation defects suggest that descendens hypoglossi anastomosis preserves neuromuscular coordination critical for complex tongue movements. In most circumstance, descendens hypoglossi provides abundant axonal input to maintain intrinsic tongue muscle tone without sacrificing hypoglossal donor fiber volume for facial reinnervation (26). While the prolonged operative time for MHFA (198.27 vs. 157.78 min, *p* < 0.001) reflects the technical complexity of dual anastomosis, the comparable facial nerve outcomes (HB improvement: 2.40 ± 0.69 vs. 2.33 ± 0.73, *p* = 0.699) and absence of additional complications confirms its safety profile.

TDI is a self-administered questionnaire we invented to evaluate tongue dysfunction (19). Notably, the strong correlation between tongue deviation angle and TDI scores (*r* = 0.890, *p* < 0.001) underscores the clinical relevance of objective deviation angle measurements in predicting subjective functional impairment, suggesting it a powerful tool in evaluating tongue dysfunction. Besides, the psychological subdomain improvement in TDI (*p* = 0.035) further highlights MHFA’s holistic benefits, addressing both physical and psychosocial sequelae of tongue dysfunction—an aspect often overlooked in surgical outcome measures.

Several limitations should be emphasized. First, the retrospective, nonrandomized, single-center design introduced chronological, learning-curve, indication, and anatomical selection biases. Conventional HFA was performed earlier, whereas MHFA was introduced later and depended on the anatomical suitability of the descendens hypoglossi. Temporal improvements may have favored MHFA, while its early learning curve may have biased outcomes unfavorably. Although multivariable models adjusted for measured covariates, residual confounding remains, and the direction and magnitude of these biases cannot be determined. Second, the outcome hierarchy was established during revision rather than prospectively registered, and secondary analyses should be considered exploratory. The study-specific tongue dysfunction score has not been formally validated, and its measurement properties and minimal clinically important difference are unknown. Tongue deviation measurements were effort- and image-dependent, while tongue pressure testing, electromyography, formal speech and swallowing assessments, and targeted evaluation of the infrahyoid muscles were unavailable. Therefore, subtle functional impairment or subclinical denervation cannot be excluded. Finally, the modest sample size and variable follow-up limited precision and long-term assessment; the absence of a significant difference in facial recovery should not be interpreted as evidence of equivalence or noninferiority.

## 5 Conclusion

MHFA represents a functionally integrative approach to facial reanimation, which was associated with less tongue dysfunction than conventional HFA. However, the retrospective design and outcome-measure limitations preclude conclusions regarding a clinically meaningful advantage. The definite advantages of MHFA still need prospective validation with standardized outcomes.

## Data Availability

The original contributions presented in the study are included in the article/supplementary material, further inquiries can be directed to the corresponding authors.

## References

[1] Holland NJ Bernstein JM. Bell's palsy. BMJ Clin Evid. (2014) 2014 (In eng) PMC3980711 24717284

[2] Khan AJ Szczepura A Palmer S Bark C Neville C Thomson D . Physical therapy for facial nerve paralysis (bell's palsy): an updated and extended systematic review of the evidence for facial exercise therapy. Clin Rehabil. (2022) 36:1424–49. (In eng). doi: 10.1177/02692155221110727, 35787015 PMC9510940

[3] Kim SJ Lee HY. Acute peripheral facial palsy: recent guidelines and a systematic review of the literature. J Korean Med Sci. (2020) 35:e245. (In eng). doi: 10.3346/jkms.2020.35.e245, 32743989 PMC7402921

[4] Ottaiano AC Gomez GD Freddi TAL. The facial nerve: anatomy and pathology. Semin Ultrasound CT MR. (2023) 44:71–80. (In eng). doi: 10.1053/j.sult.2022.11.005, 37055142

[5] Samii M Alimohamadi M Khouzani RK Rashid MR Gerganov V. Comparison of direct side-to-end and end-to-end hypoglossal-facial anastomosis for facial nerve repair. World Neurosurg. (2015) 84:368–75. (In eng). doi: 10.1016/j.wneu.2015.03.029, 25819525

[6] Jandali D Revenaugh PC. Facial reanimation: an update on nerve transfers in facial paralysis. Curr Opin Otolaryngol Head Neck Surg. (2019) 27:231–6. (In eng). doi: 10.1097/moo.0000000000000543, 31169528

[7] Kleijwegt MC Wever C Hensen EF Jansen JC Koot RW Malessy MJA. Reestablishment of the smile after hypoglossal-facial nerve transfer: what can we learn? J Neurol Surg B Skull Base. (2024) 85:546–52 (In eng). doi: 10.1055/a-2128-5191, 39228891 PMC11368455

[8] André Texeira Iora M Rodrigues Teixeira Netto M Porto Cardoso C Rossi dos Santos P Iserhardt Ciochetta C Moreira Monteiro J . Effectiveness of hypoglossal-facial anastomosis in the rehabilitation of facial paralysis following vestibular schwannoma surgery: a systematic review. Cureus. (2024) 16:e57625. doi: 10.7759/cureus.57625, 38707182 PMC11069456

[9] Dalla Toffola E Pavese C Cecini M. Hypoglossal-facial nerve anastomosis and rehabilitation in patients with complete facial palsy: cohort study of 30 patients followed up for three years. Funct Neurol. (2014) 29:183–7. (In eng) PMC4264785 25473738

[10] Zumbusch F Schlattmann P Guntinas-Lichius O. Facial nerve reconstruction for flaccid facial paralysis: a systematic review and meta-analysis. Front Surg. (2024) 11:1440953. (In eng). doi: 10.3389/fsurg.2024.1440953, 39104714 PMC11298393

[11] Negley KJ Rasool A Byrne PJ. Motor relearning after hypoglossal-facial nerve anastomosis. Am J Phys Med Rehabil. (2021) 100:e85–8. (In eng). doi: 10.1097/phm.0000000000001562, 32796157

[12] Socolovsky M Martins RS di Masi G Bonilla G Siqueira M. Treatment of complete facial palsy in adults: comparative study between direct hemihypoglossal-facial neurorrhaphy, hemihipoglossal-facial neurorrhaphy with grafts, and masseter to facial nerve transfer. Acta Neurochir. (2016) 158:945–57. (In eng). doi: 10.1007/s00701-016-2767-7, 26979182

[13] Hayashi A Nishida M Seno H Inoue M Iwata H Shirasawa T . Hemihypoglossal nerve transfer for acute facial paralysis. J Neurosurg. (2013) 118:160–6. (In eng). doi: 10.3171/2012.9.Jns1270, 23101452

[14] Tayebi Meybodi A Moreira LB Zhao X Belykh E Lawton MT Eschbacher JM . Using the post-Descendens hypoglossal nerve in hypoglossal-facial anastomosis: an anatomic and histologic feasibility study. Operative Neurosurg. (2020) 19:436–43. doi: 10.1093/ons/opz408, 31943073

[15] Conley J Baker DC. Hypoglossal-facial nerve anastomosis for reinnervation of the paralyzed face. Plast Reconstr Surg. (1979) 63:63–72. (In eng) 10.1097/00006534-197901000-00011 432325

[16] Schipper J Arndt S Maier W Spetzger U Ridder GJ. Paralyzed face. Ansa-cervicalis-nervi-hypoglossi. Chirurg. (2005) 76:47–53. (In ger). doi: 10.1007/s00104-004-0883-z, 15205750

[17] Wang X Chen G Song G Liang J Bao Y Li M. The posterior belly of digastric muscle as the landmark in facial nerve anastomosis surgery: anatomical study and case illustration. World Neurosurg. (2023) 172:e599–604 (In eng). doi: 10.1016/j.wneu.2023.01.095, 36720346

[18] Cai X Wang B Ying T Qian M Li S. Modified hypoglossal-facial nerve anastomosis for peripheral-type facial palsy caused by pontine infarction: a case report and literature review. Heliyon. (2023) 9:e16909. (In eng). doi: 10.1016/j.heliyon.2023.e16909, 37332903 PMC10275953

[19] Tang Y Chen Z Zhu J. Salvaging the transected hypoglossal nerve using descendens hypoglossi in patients undergoing hypoglossal-facial nerve anastomosis for facial palsy: a randomized clinical trial. J Neurosurg. (2024) 14:1578–86. doi: 10.3171/2024.4.JNS232971 39059421

[20] Catli T Bayazit YA Gokdogan O Goksu N. Facial reanimation with end-to-end hypoglossofacial anastomosis: 20 years' experience. J Laryngol Otol. (2010) 124:23–5. (In eng). doi: 10.1017/s0022215109991344, 19785928

[21] Altamami NM Zaouche S Vertu-Ciolino D. A comparative retrospective study: hypoglossofacial versus masseterofacial nerve anastomosis using Sunnybrook facial grading system. Eur Arch Otorrinolaringol. (2019) 276:209–16. (In eng). doi: 10.1007/s00405-018-5186-y, 30377760

[22] Hamdi OA Jones MK Ziegler J Basu A Oyer SL. Hypoglossal nerve transfer for facial nerve paralysis: a systematic review and Meta-analysis. Facial Plast Surg Aesthet Med. (2024) 26:219–27. (In eng). doi: 10.1089/fpsam.2023.0144, 38153410

[23] Fík Z Kraus J Čada Z Chovanec M Fíková A Zvěřina E . Hemihypoglossal-facial nerve anastomosis: results and electromyographic characterization. Eur Arch Otorrinolaringol. (2022) 279:467–79. (In eng). doi: 10.1007/s00405-021-06893-4, 34036422

[24] Slattery WH 3rd Cassis AM Wilkinson EP Santos F Berliner K. Side-to-end hypoglossal to facial anastomosis with transposition of the intratemporal facial nerve. Otol Neurotol. (2014) 35:509–13. (In eng). doi: 10.1097/MAO.0b013e3182936bcf, 24518409

[25] Martins RS Socolovsky M Siqueira MG Campero A. Hemihypoglossal-facial neurorrhaphy after mastoid dissection of the facial nerve: results in 24 patients and comparison with the classic technique. Neurosurgery. (2008) 63:310–7. doi: 10.1227/01.NEU.0000312387.52508.2C, 18797361

[26] Pitty LF Tator CH. Hypoglossal-facial nerve anastomosis for facial nerve palsy following surgery for cerebellopontine angle tumors. J Neurosurg. (1992) 77:724–31. (In eng) 10.3171/jns.1992.77.5.0724 1403114

